# Assessment of seat belt use and its associated factors among public transport drivers in North Gondar, Ethiopia: a cross-sectional study

**DOI:** 10.1186/s13104-019-4140-4

**Published:** 2019-02-22

**Authors:** Manay K. Woldegebriel, Berihu G. Aregawi, Hafte T. Gebru

**Affiliations:** 1 Department of Public Health, College of Health Sciences and Referral Hospital, Aksum University, Aksum, Ethiopia; 2 Department of Biomedical Sciences, College of Health Sciences and Referral Hospital, Aksum University, P.O.Box: 298, Aksum, Ethiopia

**Keywords:** Drivers, Ethiopia, Knowledge, Seat belt use

## Abstract

**Objective:**

Road traffic injuries are the major and neglected public health challenges. It causes 1.2 million deaths and 50 million injuries yearly and the use of seat belt reduces 60% of the cases. However, little is known about the magnitude of utilizing seat belt and associated factors in Ethiopia. Hence, the aim of this study was to assess the seat belt practice and associated factors among minibus and taxi drivers.

**Results:**

The magnitude of seat belt users is 69.6%. The majority (98.1%) of drivers used seat belt to minimize injuries, 95.8% to prevent casualties, 92.5% to safeguard vehicle occupants, 29.9% to generate revenue for government and 22.8% to beautify the vehicle. Almost 80% of participants reported that wearing seat belt could save lives; and 29.6% of them wear belts because of stiffer penalties. For not using seat belts, more than 18% drivers reasoned out that it is not guarantee for safety and it wastes time to wear. In the multiple logistic regression being taxi driver (AOR = 1.998, 95% CI 1.250, 3.192), being married (AOR = 2.91, 95% CI 1.118, 7.601) and attended vocational school and above (AOR = 2.140, 95% CI 1.014, 4.519) were associated with seat belt use.

## Introduction

Road traffic injuries are a major and neglected public health challenge that requires intensive efforts for effective and sustainable prevention. According to the World Health Organization (WHO) report, 1.2 million peoples die and 50 million get injured from road traffic crashes every year [1]. It will be the third leading contributor to the global burden of disease and injury by 2020 [2, 3]. Most of (91%) road deaths occurred in low and middle income countries [4].

In the case of Africa, road traffic accidents constitute 25% of all injury related deaths [5] and over 75% of road traffic casualties are in the economic productive age of 16–65 years. In Ethiopia, it is the cause of significant losses of human and economic resources. In 2014/2015 Ethiopian police reported 15,086 accidents which caused the losses of 2161 lives and over US$ 7.3 million. The Ethiopian National Road Safety Coordination Office cites a road crash fatality rate of 114 deaths per 10,000 vehicles per year and the real figure may be higher as there may be underreporting [6]. Study done in Ethiopia, on the road from Addis Ababa to Adama/Hawassa showed that the fatality rate of car accident was 156 per 10, 1000 vehicles [7]. A study in Amhara region between 2007 and 2011, shows that there were 10,162 road traffic accidents, claiming the lives of 2761 people, injuring 3890 people and caused property damage of 4,755,514 USD [8].

Injury control is a public health problem and we have an ethical responsibility to arrange for the safety of individuals [9]. Seat belt use prevents and reduces the severity of injuries during motor vehicle crashes [10, 11]. Mandatory seat belt laws, their enforcement and appropriate public awareness campaigns have been shown to be very effective in increasing rates of seat belt wearing [4]. Reducing stress, enhancing psychological and physiological health status greatly reduced the road traffic accidents. Physical exercise was also important to improve safety [12]. The work related stress and smoking affected the life style of drivers and it was significantly associated with the safety of road traffic among drivers [13].

There are different observations [14–20] and self-reported studies [10, 21–25] on seat belt use out of Ethiopia. But in the case of Ethiopia, little was known about the use of the seat belt and factors that affect using seat belt among drivers. So, the aim of this study was to assess the magnitude of seat belt use practice and associated factors among taxi and minibus drivers.

## Main text

### Study design and study area

Cross-sectional study was employed to assess seat belt use and associated factors among drivers in North Gondar Zone. In 2016 there were 453 mini buses and 282 taxies registered under the North Gondar Road & Transport Authority.

### Sampling technique

The list of minibus was taken from North Gondar Road and Transport office and list of taxies from two taxi associations. Then, sampling frame was prepared and simple random method was employed for selection. Total of 262 minibus and 163 taxi drivers were selected for data collection.

### Data processing and analysis

Data were entered into Epinfo version 3.5.1 and exported to SPSS for analysis. Findings are summarized by tables and texts. Bivariable analysis was done and variable with P ≤ 0.2 were entered to multiple logistic regression analyses. The strength of association was determined using odds ratio at 95% confidence interval and P < 0.05; the Hosmer–Lemeshow statistic was used to check the model of goodness.

### Results

#### Socio-demographic and behavioral characteristics of drivers

A total of 425 drivers were participated in the study of which 38.4% were taxi drivers and 61.6% minibus drivers and there was only one female participant. About 86% of the drivers were Christians and the rest (13%) are Muslims. More than half (56.7%) of the drivers served for 5 years and above; and the rest (43.3%) served for less than 5 years in deriving. Most of the drivers (55.3%) were aged 25–30 years and 17.2%, 15.5%, and 12% of the participants were aged 31–36, 19–24 and ≥ 37 years respectively. With regard to the educational status, 37.2% of the drivers completed grades 9–10, 25.2% completed grades 11–12 and about 20% of them took vocational training and above. The number of participants who were married and single was comparable. There were 91 (21.4%) drivers who use alcohol and the rest (78.6%) were non-alcoholic.

#### Knowledge of drivers about the use of seat belts

The knowledge questions were scored, with a mean score of 5.82 ± 1.22. The respondents scored mean and above (5–7 points) were considered as having a good knowledge and below the mean (0–4 points) was considered as having poor knowledge. About 61.4% of respondents have good knowledge of using seat belt while the remaining 38.6% respondents score poor knowledge.

The majority of the drivers (98.1%) thought the Ethiopian seat belt law had been made to minimize injuries, 95.8% said to prevent casualties, 92.5% said to safeguard vehicle occupants, 29.9% said to generate revenue for the government and 22.8% to beautify the vehicle while 2.8% of drivers did not know about the law. Concerning the category of people who must use seat belts; 76.0% drivers said everybody should use, 9.2% said drivers only, 14.6% said drivers and front sitters and 0.2% of them said passengers only. Almost comparable number of participants reported that seat belt should be fastened before and after the engine is ignited (Table 1).

**Table 1:** Knowledge of minibus and taxi drivers regarding the seat belt use, Gondar, Ethiopia, 2016

Knowledge questions	N (%)
Reasons for setting seat belt law	
To minimize injuries	
Yes	417 (98.1)
No	8 (1.9)
To prevent casualties	
Yes	407 (95.8)
No	18 (4.2)
To safeguard vehicle occupants	
Yes	393 (92.5)
No	32 (7.5)
To generate revenue for government	
Yes	127 (29.9)
No	298 (70.1)
To beautify the vehicle	
Yes	97 (22.8)
No	328 (77.2)
Do not know about the law	
Yes	12 (2.8)
No	413 (97.2)
Category of people to use safety belt	
Everybody/passengers	323 (76.0)
Drivers only	39 (9.2)
Drivers and front sitters	62 (14.6)
Passengers only	1 (0.2)
The right time to fasten safety belt	
Before igniting the engine	219 (51.5)
After igniting the engine	191 (44.9)
While driving on the road	15 (3.5)
Before igniting the engine	219 (51.5)

#### Self-reported seat belt use practice

The possible responses for “how often do you wear a seat belt when driving a car?” were, never wear seat belt, sometimes, most of the time and always. For the purpose of analysis, “most of the time and always” were denoted as “users” and never and sometimes were grouped as “non users” and finally, 69.6% were users and 30.4% were non users.

Type of car, marital status, educational level and alcohol drinking were associated (P ≤ 0.2) with seat belt use in bivariable analysis and transferred to multiple logistic regression. However, type of car, marital status, educational level remained significant (P < 0.05). Taxi drivers were more likely to use seat belt than minibus drivers, it is about two times higher (AOR = 1.99, 95% CI 1.25, 3.19). Married drivers were almost three times more likely to use seat belts compared to separated and cohabited drivers (AOR = 2.92, 95% CI 1.12, 7.60) and drivers who attended vocational school and above were 2.14 times higher to use seat belt than those who attended grade eight and below (AOR = 2.14, 95% CI 1.01, 4.52) (Table 2).

**Table 2:** Bivariable and multple logistic regression analyses of factors associated with seat belt use among minibus and taxi drivers, Gondar, Ethiopia, 2016

Variable	Seat belt use		CR (95% CI)	P-value	AOR (95% CI)	P-value
	Users	Non users				
Car type						
Taxi	129	34	2.16 (1.37, 3.39)	0.001	1.99 (1.25, 3.19)	0.004
Minibus	167	95				
Marital status						
Married	138	52	2.65 (1.04, 6.75)	0.040	2.92 (1.12, 7.60)	0.029
Single	148	67	2.21 (0.88, 5.56)	0.092	2.29 (0.89, 5.95)	0.087
Others^a^	10	10		0.116	1.00	0.080
Age (in years)						
19–24	51	15	1.00	0.099		
25–30	155	80	0.57 (0.30, 1.08)	0.083		
31–36	57	16	1.05 (0.47, 2.33)	0.909		
≥ 37	33	18	0.54 (0.24, 1.22)	0.137		
Education						
≤ Grade 8	49	27	1.00	0.016	1.00	0.059
Grade 9–10	100	58	0.95 (0.54, 1.68)	0.860	0.914 (0.51, 1.64)	0.762
Grade 11–12	78	29	1.48 (0.79, 2.79)	0.224	1.41 (0.74, 2.69)	0.303
Vocational and above	69	15	2.54 (1.22, 5.26)	0.012	2.14 (1.01, 4.52)	0.046
Alcohol drinking						
Yes	69	22	1.00			
No	227	107	0.68 (0.39, 1.15)	0.150		
Duration of driving service (years)						
< 5	130	54	1.00			
≥ 5	166	75	0.92 (0.61, 1.39)	0.694		

^a^Separated and cohabited

#### Reasons for using and not using seat belt by the drivers

Drivers who were using seat belt were requested to forward their reasons for using seat belts. Most of (79.3%) the drivers said that seat belts could save lives, 4.2% stated that they wear seat belt because they simply heard it from mass media promotion, 29.6% reasoned that the presence of stiffer penalties for non-compliance with the seat belt law, 4% because there is an alarm system in the car and 5.9% of them were wearing seat belts as it was habit for them. Similarly, drivers who didn’t use seat belt were asked why and not believing in seat belt safety (18.4%), wasting time to wear seat belt (18.40%) and discomforts with the seat belts (7.80%) were the most common reasons for not using seat belt (Table 3).

**Table 3:** Self reported reasons for using and not using seat belt among minibus and taxi drivers, Gondar, Ethiopia, 2016

Reasons for not using seat belt	Frequency (%)	Reasons for using seat belt	Frequency (%)
Frequent stops	1.40	Mass media promotion	4.20
Forgetting	1.40	Penalty for non compliance	29.6
It is norm in our culture	3	Saves life	79.30
Driving slowly	5.20	Alarm system is there in the car	4
Creates discomfort	7.80	It is habit	5.90
Not believe of its safety	18.40		
Takes time to wear	18.4		

### Discussion

The use of seat belt in the current study was only 69.6% which is relatively similar to studies in Russia (55%) [24] and USA (59.0%) [21]. The seat belt use rate is higher than studies on taxi drivers of Beijing (7.7%) [17], Thailand (28.46%) [20], Nigeria (18.9%) [19], West Indies (31.6%) [26] and Armenia (24%) [22]. However, it was lower than a study among vehicle drivers in Nigeria (80%) [23]. Using seat belts in North Gondar is lower, which has serious implications on safety so significant effort must be made to improve the use of seat belts in order to reduce morbidity and mortality from injuries. By effectively coupling media and enforcement campaigns, significant increase in seat belt usage must be achieved. The media-based approach for education and outreach on the use of seat belts was effective in increasing the public’s awareness of the campaign in Nevada [27]. Therefore, different training strategies at bus stations and bus stops should be designed and applied to promote and/or encourage the use of seat belt.

In the current study, both age and drinking alcohol didn’t predict the practice of seat belt use. This might be because of the nature of the study; difference in sampling, sample size and/or the difference on how participants perceive drinking alcohol. Alcohol impairs the reaction time of drivers and their ability to estimate risks and it is considered to be serious violation of traffic law [28] and finding from Thiland and America showed that seat belt use rate among drinkers was lower [21, 29, 30]. In addition, another American study found that older drivers were more likely to use belts compared to young drivers [31] and similarly, in the case of Ontario seat belt use increased with aging [32]. Regarding aging, the current finding was consistent with reports from West Indies [33], Nigeria [15], Russia [24] and Thai [20].

There married drivers used seat belt more than separated and cohabited drivers (AOR = 2.915, 95% CI 1.118, 7.601). This is comparable with finding from West Indian study [33]. This can be explained by the fact that the married ones have additional responsibilities for their families and more likely to respect road safety measures and precautions.

In this study, more taxi drivers were using seat belts than bus drivers did (AOR = 1.998, 95% CI 1.250, 3.192). Studies elsewhere also found similar reports; in Nigeria [15], Nanjing and Zhoushan, China [15] and Nanjing, China [18] taxi drivers were more likely to use seat belts compared to bus and pickup drivers. Traffic laws are less emphasized in roads outside cities due to limited resources to control [34]. In the same way, in Utah drivers in urban locations and those driving on the interstate were more likely to wear seat belt [14] and also Malaysian car drivers in city center areas were more likely to wear seat belts compared to those driving outside city [34].

Many findings from various studies showed that educational level of drivers was significantly associated with using seat belt. Drivers who attended vocational school and above used seat belt more than those who attended grade eight and lower (AOR = 2.140, 95% CI 1.014, 4.519). Reports from West India [33] and Northwestern Nigeria showed that educational level was positively and significantly associated with wearing seat belt [26]. In USA, education was also markedly associated with using seat belt [35]. The compliance level of wearing belts increased with the level of education in Malaysian study [34]. This is worthy to consider as education always contributes to a positive change. The current finding contradicts with study from Russia where college degree drivers were found to have a significant negative association with using seat belt [24].

In the current study, common reasons for wearing seat belt were, “seat belt saves life” (79.3%), “stiffer penalties for non-compliance” (29.6%) and “wearing habit” (5.9%). These reasons were comparable to reports from China [25], Russia [29], Quatar [36] and West India [33]. In many studies, drivers did not like seat belts because of discomfort, lack of knowledge, not believing in seat belt safety, forgetfulness and habit to wear were reported [25, 31, 33, 36]. In concordance with the above studies, in the current study the main reasons given for lower use were “not believing in seat safety” (18.4%), “seat belt takes time to wear” (18.4%) and “creates discomfort” (7.8%). These findings suggest that we still need promotion on using seat belt during licensing and on job training to enhance safety.

### Conclusion

Despite the proven effectiveness of seat belt, its use was lower (69.6%) in northern Gondar. The type of car, marital status and drivers’ level of education were significantly associated with the practice of seat belt. Therefore, drivers should be encouraged to use seat belts when they are at work and during licensing.

### Limitation of the study

As this is a self reported data, participants may over estimate themselves on using seat belts. The method of data collection is open to self-reported and social desirability bias that may affect the result. It is also difficult to appreciate gender based comparisons and a discussion as only one female participant was included in the sample.

## Abbreviations

AOR: adjusted odds ratio
CI: confidence interval
COR: crude odds ratio
SPSS: Statistical Package for the Social Science
